# Impact of malnutrition on systemic immune and metabolic profiles in type 2 diabetes

**DOI:** 10.1186/s12902-020-00649-7

**Published:** 2020-11-12

**Authors:** Anuradha Rajamanickam, Saravanan Munisankar, Chandra Kumar Dolla, Kannan Thiruvengadam, Subash Babu

**Affiliations:** 1 National Institute of Health-NIRT-International Center for Excellence in Research, Chetpet, Chennai, 600031 India; 2 National Institute for Research in Tuberculosis, Chennai, India; 3 Laboratory of Parasitic Diseases, National Institute of Allergy and Infectious Diseases, National Institutes of Health, Bethesda, MD USA

**Keywords:** Malnutrition, Type 2 diabetes mellitus, Adipocytokines, Pancreatic hormones, Cytokines

## Abstract

**Background:**

While obesity and overweight status are firmly established risk factors for Type 2 diabetes mellitus (T2DM), a substantial proportion of diabetic individuals, especially in Africa and Asia, are often underweight or normal weight. However, very little is known about the immunological and metabolic profiles of these individuals.

**Methods:**

This study aimed to assess the relationship between malnutrition and Type 2 diabetes mellitus (T2DM). We examined a variety of analytes associated with the immunological and metabolic profiles of T2DM individuals with low (< 18.5 kg/m2) or normal (18.5–24.9 kg/m2) body mass index (BMI). To this end, we measured plasma levels of HbA1c, glucose, insulin, glucagon, adipocytokines and Type 1, Type 2, Type 17, pro-inflammatory and regulatory cytokines in T2DM individuals with low BMI (LBMI) or normal BMI (NBMI) with small sample size *n* = 44 in each group.

**Results:**

LBMI individuals exhibited significantly higher levels of HbA1c, random blood glucose, insulin and glucagon compared to NBMI individuals. Similarly, LBMI individuals exhibited significantly higher levels of adiponectin and adipsin and significantly lower levels of leptin in comparison to NBMI individuals. LBMI individuals also exhibited significantly lower levels of the Type 1, Type 2, Type 17, pro-inflammatory and regulatory cytokines in comparison to NBMI individuals. Finally, while the metabolic parameters exhibited a significant negative correlation with BMI, the immunological parameters exhibited a significant positive correlation with BMI.

**Conclusions:**

Malnutrition is associated with a significant modulation of glycemic, hormonal and cytokine parameters in T2DM. Hence, the biochemical and immunological profiles of T2DM is significantly influenced by BMI.

**Supplementary Information:**

The online version contains supplementary material available at 10.1186/s12902-020-00649-7.

## Background

Globally, there are an estimated 425 million people living with T2DM (Type 2 Diabetes Mellitus) and the prevalence is projected to increase to 629 million by 2045 [1]. The highest increase in prevalence is expected to occur in low and middle-income countries (LMIC), where malnutrition is co-prevalent [1]. While obesity and overweight status are known risk factors for development of T2DM, there is emerging evidence that T2DM also occurs in individuals in normal or underweight individuals, especially in LMIC [2–4]. A recent analysis reported that the prevalence of T2DM in normal or underweight individuals ranged from 1.4 to 8.8%, which was not very different from that in the general population (1.4 to 10.9%) [2]. In addition, in Asia and Africa, the proportion of individuals with diabetes, who were of normal or low BMI (Body Mass Index), ranged from 24 to 66% [2]. It has been proposed that impairment in insulin secretion, in utero undernutrition and epigenetic alterations to the genome might contribute to the pathogenesis of diabetes in the low BMI group [5]. Acute or chronic diseases and its treatment interference could also result in the exacerbation of malnutrition, mainly undernutrition, due to alteration in metabolism [6]. Malnutrition (undernutrition) in those with diabetes leads to impaired muscle function and wound healing, decreased bone mass, immune dysfunction, and general functional decline [7, 8].

Globally, malnutrition is a severe public health issue with a prevalence of 925 million globally [9–11]. Malnutrition is a clinical disorder that comprises a range of anthropometric deficiencies from reduce (low weight for height) and impede (low height for age) in undernutrition to other disorders of nutrition including high BMI such as overweight and obesity [11, 12]. Undernutrition/malnutrition is typically defined by the presence of both low BMI and low serum albumin levels [13]. It is associated with a variety of metabolic abnormalities, including steatosis, increased lipolysis and fatty acid oxidation, decreased circulating amino acids, decreased peroxisome number and function and impaired mitochondrial function [13, 14]. Malnutrition can lead to immune dysfunction and enhanced mortality from infections [15–17].

We hypothesized that malnutrition could alter pancreatic hormone, adipocytokine and cytokines response in T2DM individuals and thereby predispose individuals to an increased risk for more severe form of T2DM. Since there is a paucity of information about the link between malnutrition and metabolic diseases of inflammatory origin, we studied the relationship between undernutrition and T2DM and assessed the influence of malnutrition on factors which are essential in glycemic control. Previous studies have shown that adipocytokines play a crucial role in T2DM [18]. Therefore, in the present study we wanted to examine the associations of circulating levels of HbA1c (Glycosylated haemoglobin), blood glucose, pancreatic hormones (insulin and glucagon) and adipocytokines (adiponectin, adipsin, resistin, leptin and visfatin). Finally, we extend the analysis of the role of inflammation in T2DM by measuring the levels of an array of cytokines in those with T2DM with or without coexisting low BMI.

## Methods

### Ethics statement

All procedures performed in studies involving human participants were in accordance with the ethical standards of the institutional and/or national research committee and the natural history study protocol (12-I-073) approved by Institutional Review Boards of the National Institute of Allergy and Infectious Diseases (USA) and the National Institute for Research in Tuberculosis (India) Ethical committee approval number NIRT-IEC ID: 2013001. Before getting informed consent, the health care provider explains about the risks, benefits, the nature of the procedure, reasonable alternatives, risks and benefits of alternatives, and assessment of the participants understanding of above mentioned factors.

### Study population

**Fig. 1:**
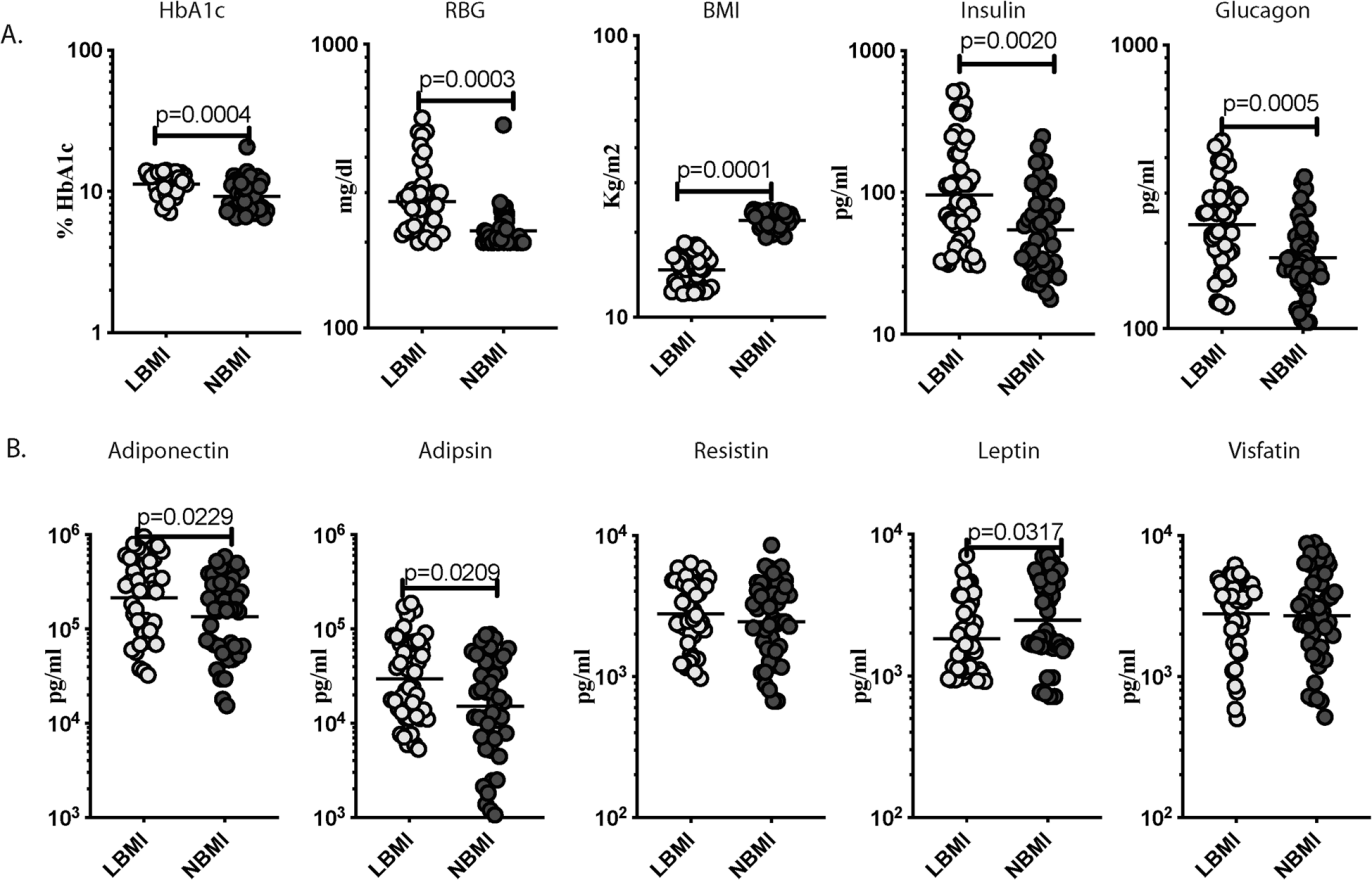
Higher circulating levels of glycemic parameters, pancreatic hormones and leptin in LBMI individuals with T2DM. **a** Plasma levels of glycated haemoglobin (HbA1c), random blood glucose (RBG), insulin and glucagon in LBMI (*n* = 44) and NBMI (*n* = 44) individuals with T2DM. **b** Plasma levels of adiponectin, adipsin, resistin, leptin and visfatin in LBMI (*n* = 44) and NBMI (*n* = 44) individuals with T2DM. Each dot represent a single participant and the bar indicating the geometric mean (GM). Mann– Whitney U-test with Holms correction for multiple comparisons were done by *p*-values are multiplied by the number of comparisons

We enrolled small sample size of 88 study participants with T2DM, of which 44 individuals were with LBMI (Low Body Mass Index) and 44 individuals were with NMBI (Normal Body Mass Index) from Kanchipuram District, Tamil Nadu, South India (Table 1). Participant recruitment flowchart was shown in S.Figure.1 Samples were obtained from all the participants. No power calculations were done to determine sample size and it was based on samples available following matching for age and sex. All the participants were screened for diabetes and nutritional indices. Participants of both gender and age between 18 and 75 years were included. Study participants having liver disease, renal disease, cardiac disease, respiratory disease or any other acute or chronic diseases, HIV, hypertension, cancer and any previous history of anthelmintic treatment or a history of helminth infections were precluded from the study. Pregnant women were also excluded from the study. Based on 2013 American Heart Association/ American College of Cardiology guidelines (LBMI< 18.5 and NBMI between 18.5 and 24.9 kg/m^2^), Low and normal BMI were defined [19]. In addition, undernutrition was confirmed by the presence of low serum albumin (< 3.4 g/dl) in all the LBMI individuals [11]. BMI was calculated as body weight (in kilograms) divided by body height (in meters) squared.

**Table 1:** Demographic and biochemical parameters of the study population

	LBMI (**n** = 44)	NBMI (***n*** = 44)	***P*** Value
**M/F**	22/22	22/22	NS
**Age**	37 (23–55)	39 (24–62)	NS
**Body mass index (Kg/m**^**2**^**)**	16.58 (12.18–18.32)	23.20 (20.93–24.00)	*p* = 0.0001
**Albumin (g/dl)**	3.1 (2.6–3.3)	4.2 (3.6–4.6)	*p* < 0.001
**Urea (mg/dl)**	22 (12–49)	20 (13–47)	NS
**Creatinine (mg/dl)**	0.89 (0.7–1.2)	0.85 (0.6–1.1)	NS
**Alanine Amino Transferase (U/L)**	24 (12–92)	22 (14–57)	NS
**Aspartate Amino Transferase (U/L)**	30.1 (14–110)	28.7 (12–68)	NS
**Serum triglycerides (mg/dl)**	117.1 (63–485)	102 (64–424)	NS
**High density lipoprotein cholesterol (ml/dl)**	39.1 (18.9–75.5)	37 (29–59)	NS
**Low density lipoprotein cholesterol (ml/dl)**	105.8 (48–182)	104 (44–180)	NS

The values represent geometric mean and range except for gender and age (which is median and range) **LBMI** refers to individuals with low BMI with T2DM, **NBMI** refers to individuals with normal BMI with T2DM. *NS* Not significant. Non parametric Mann-Whitney U tests was used to calculate the difference between the two groups

### Anthropometric measurements

An ACT 5 Diff. hematology analyzer (Beckman Coulter, Brea, CA, USA) was used to quantify haematological parameters using venous EDTA blood samples. Anthropometric assessments (comprising height, weight and waist circumference) were assessed by trained personnel [20].

### Biochemical analysis of blood samples

Overnight fasting venous blood samples were collected from the recruited individuals in EDTA -containing tubes using standardised protocol and equipment. They were separated into two samples: the first sample containing whole blood for the measurement of HbA1c and the other plasma specimen was used for lipid profile levels and renal and liver function tests. Random Blood Glucose (RBG) test was done within two hours of eating and analysed using auto analyser. The RBG normal value should be 180 mg/dl as per the American Diabetes Association. Plasma samples were used to measure other biological parameters [2, 21].

### Determination of T2DM status

Based on American Diabetes Association criteria, Type 2 diabetes was confirmed by an HbA1c value of 6.5% or greater and a random blood glucose of > 200 mg/dl. Overnight fasting samples were used to determine all biochemical parameters with exclusion of random blood glucose [2, 21]. All diabetic participants were newly diagnosed, not on any anti-diabetic medication at the time of blood draw and without any known complications or co-morbidities. All participants were referred to the primary health care centre for diabetic treatment.

### Measurement of plasma adipocytokines and cytokine levels

Pancreatic hormones (insulin and glucagon), adipocytokines (adiponectin, adipsin, resistin, leptin, visfatin) and the levels of Type 1 cytokines: IFNγ, TNFα, IL-2, Type 17 cytokines: IL-17A, IL-22, Type 2 cytokines: IL-4, IL-5, IL-13, regulatory cytokine: IL-10 and pro-inflammatory cytokines: G-CSF, GM-CSF, MCP-1, MIP-1β, IL-6, IL-7, IL-8, IL-12p70 and IL-1β plasma levels were estimated using Bioplex multiplex assay system (Bio-Rad, Hercules, CA). TGF-β and IL-17F were measured by conventional ELISA according to the manufacturer’s protocol (R&D Systems, Minneapolis, MN, USA). All the measured variables such as HbA1c, cytokines and hormones were illustrated as mean, SD, intra-assay coefficient of variation (CV%), inter-assay coefficient of variation (CV%) and detection limit were shown in Table S1.

### Statistical analysis

Values were expressed as geometric means. Shapiro-Wilks test was used to assess normality of the data. Nonparametric Mann-Whitney U test was used to compare the pro-inflammatory and regulatory cytokines between the group LBMI and NMBI. Spearman rank correlation was performed to assess the relationship of the BMI with glycemic parameters, pancreatic hormones, adipocytokines. Appropriate power calculation was done to assess the acceptability of the statistical findings.

Univariate and Multiple logistic regression models were used to measure the association between various factors and Low Body mass Index. Odds ratio and 95% CIs were reported for each factor in the univariate and for the factors adjusting for age and gender in the final multivariate models. Principle Component Analysis (PCA) was used with a goal of identifying potentially significant patterns of pancreatic hormones, adipocytokines and cytokines which is responsible for any of the clustering/separation between the normal and low body mass index.

JMP14 software was used to plot Principle Component Analysis (PCA). Further data analysis was performed using STATA 15.0 (StataCorp, College Station, TX). Graphical representation was made using Graph-Pad PRISM Version 8.0 (GraphPad, San Diego, CA). All *p*-values were two-sided with statistical significance evaluated at the 0.05 alpha level.

## Results

### Study population characteristics

Study participants demographics and biochemical parameters at baseline are presented in Table 1. The median age of the LBMI group was 37 years and the age ranged from 23 to 55 years. The median age of the NBMI group was 39 years and the age ranged from 24 to 62 years. Age, sex, lipid profile or other biochemical parameters did not differ significantly between the two groups. Baseline hematology parameters are listed in Table 2. Haematological parameters did not differ significantly between two groups.

**Table 2:** Haematological parameters of the study population

	LBMI	NBMI	***P*** Value
**Haemoglobin (g/dl)**	12.4 (8.4–16.3)	13.51 (9.1–18.6)	NS
**Red blood cell count (10**^**6**^**/L)**	4.6 (3.3–5.8)	4.9 (3.7–6.2)	NS
**White blood cell count (10**^**3**^**/L)**	70 (45–112)	77 (49–117)	NS
**Haematocrit (%)**	40.1 (26.6–49.7)	40.6 (27.8–54.0)	NS
**Platelet count (10**^**3**^**/L)**	256.5 (135–154)	292.7 (162–387)	NS
**Neutrophils cells/ml**	3342 (2642–5917)	3928 (2922–6735)	NS
**Lymphocytes cells/ml**	2504 (1285–3894)	2454 (15982–4783)	NS
**Monocytes cells/ml**	430 (29–869)	497 (243–985)	NS
**Eosinophils cells/ml**	441 (89–1755)	390 (80–2718)	NS
**Basophils cells/ml**	53 (20–256)	60 (18–397)	NS

The values represent geometric mean and range; *NS* Not significant

### Higher levels of HbA1c, blood glucose, pancreatic hormones, adiponectin and adipsin but lower levels of leptin in LBMI individuals

To examine the impact of malnutrition on indices of glucose control in T2DM, we measured the levels of glycated haemoglobin (HbA1c), random blood glucose (RBG) and pancreatic hormones in LBMI and NMBI individuals. As exhibited in Fig. 1a, the levels of HbA1c (Geometric Mean (GM) of 11.27% in LBMI Vs 9.2% in NBMI), RBG (GM of 279.6 mg/dl in LBMI Vs 220.4 mg/dl in NBMI), insulin (GM of 95.88 pg/ml in LBMI Vs 54.13 pg/ml in NBMI) and glucagon (GM of 232.5 pg/ml in LBMI Vs 177.5 pg/ml in NBMI) were significantly higher in LBMI in comparison with NBMI individuals. Subsequently, we examined the impact of malnutrition on adipocytokines in T2DM by measuring the plasma levels of adiponectin, adipsin, resistin, leptin and visfatin in LBMI and NBMI individuals. As exhibited in Fig. 1b, LBMI individuals showed significantly higher levels of adiponectin (GM of 214,516 pg/ml in LBMI Vs 134,223 pg/ml in NBMI) and adipsin (GM of 29,794 pg/ml Vs 15,054 pg/ml) and in contrast, significantly lower levels of leptin (GM of 1832 pg/ml Vs 2469 pg/ml) when compared to NBMI individuals. Hence, malnutrition is connected with higher levels of the HbA1c, RBG, pancreatic hormones, adiponectin and adipsin and lower levels of leptin in T2DM individuals.

### Lower circulating levels of type 1, type 17, type 2 and regulatory cytokines in LBMI individuals

**Fig. 2:**
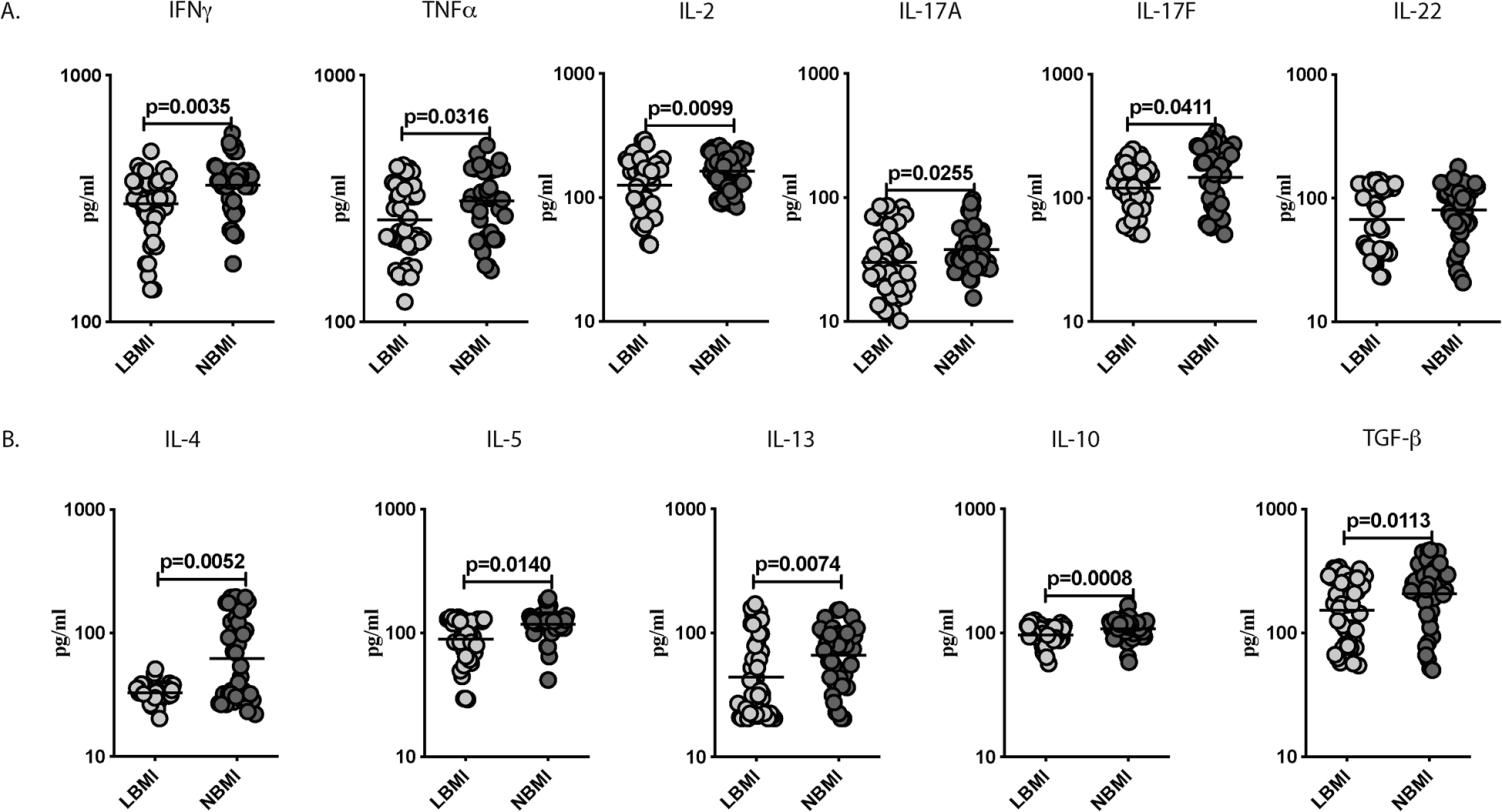
Lower circulating levels of Type 1, Type 17, Type 2 and regulatory cytokines in LBMI individuals with T2DM. **a** Plasma levels of Type 1 (IFNγ, TNFα and IL-2) and Type 17 (IL-17A, IL-17F and IL-22) cytokines in LBMI (n = 44) and NBMI (n = 44) participants with T2DM. **b** Plasma levels of Type 2 (IL-4, IL-5 and IL-13) and regulatory (IL-10 and TGF-β) cytokines in LBMI (n = 44) and NBMI (n = 44) participants with T2DM. Each dot represent a single participant and the bar indicating the geometric mean (GM). Mann– Whitney U-test with Holms correction for multiple comparisons were done by p-values are multiplied by the number of comparisons

Next, we wanted to study, whether malnutrition in T2DM was linked with changes in the levels of Type 1 and Type 17 cytokines. To this end, we measured the circulating levels of Type 1 (IFNγ, TNFα and IL-2)- and Type 17 (IL-17A, IL-17F and IL-22) cytokines in LBMI and NBMI individuals. As exhibited in Fig. 2a, LBMI individuals had significantly lower levels of IFNγ (GM of 300.9 pg/ml Vs 357.7 pg/ml), TNFα (GM of 259.5 pg/ml Vs 309.8 pg/ml), IL-2 (GM of 125.6 pg/ml Vs 163.3 pg/ml), IL-17A (GM of 30.1 pg/ml Vs 38.3 pg/ml) and IL-17F (GM of 120.6 pg/ml Vs 146.8 pg/ml) in comparison with NBMI individuals.

Further, we wanted to study, whether malnutrition in T2DM was linked with changes in levels of Type 2 and regulatory cytokines. To this end, we measured the circulating levels of Type 2 (IL-4, IL-5 and IL-13) and regulatory (IL-10 and TGF-β) cytokines in LBMI and NBMI individuals. As exhibited in Fig. 2b, IL-4 (GM of 32.8 pg/ml Vs 61.9 pg/ml), IL-5 (GM of 89.4 pg/ml Vs 117.6 pg/ml), IL-13 (GM of 43.8 pg/ml Vs 65.9 pg/ml), IL-10 (GM of 96.3 pg/ml Vs 108.2 pg/ml) and TGF-β (GM of 153.1 pg/ml Vs 208.7 pg/ml) levels were significantly lower in LBMI in comparison with NBMI individuals. Hence, malnutrition with T2DM is linked to lower circulating levels of Type 1, Type 17, Type 2 and regulatory cytokines.

### Lower circulating levels of other pro-inflammatory cytokines in NBMI individuals

**Fig. 3:**
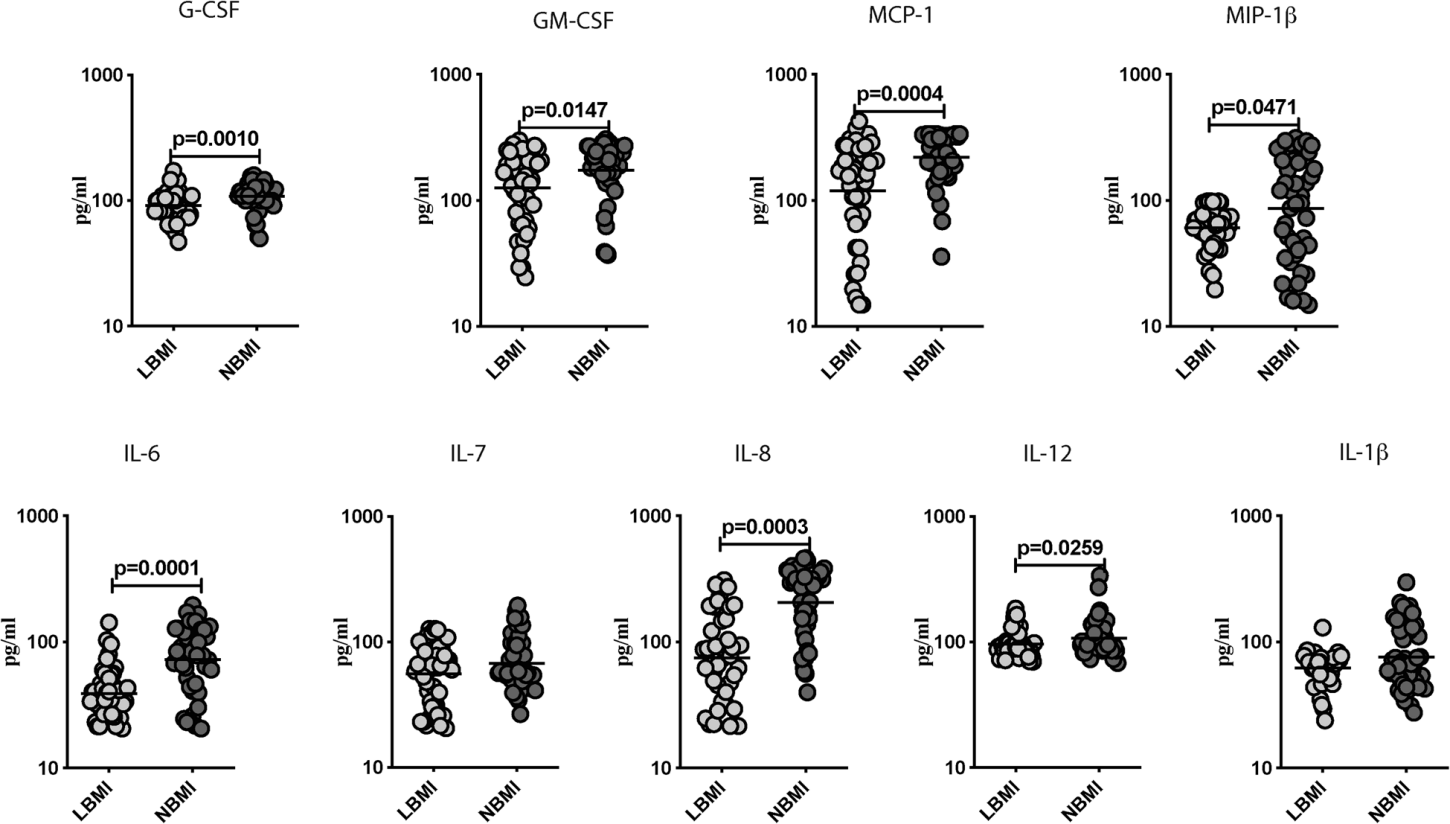
Lower circulating levels of other pro-inflammatory cytokines in LBMI individuals with T2DM. **a** Plasma levels of other pro-inflammatory (G-CSF, GM-CSF, MCP-1, MIP-1β, IL-6, IL-7, IL-8, IL-12p70 and IL-1β) cytokines in LBMI (n = 44) and NBMI (n = 44) participants with T2DM. Each dot represent a single participant and the bar indicating the geometric mean (GM). Mann– Whitney U-test with Holms correction for multiple comparisons were done by p-values are multiplied by the number of comparisons

To examine the impact of malnutrition on other pro-inflammatory cytokines in T2DM, we measured the circulating levels of pro-inflammatory cytokines (G-CSF, GM-CSF, MCP-1, MIP-1β, IL-6, IL-7, IL-8, IL-12p70 and IL-1β) in LBMI and NBMI individuals. As exhibited in Fig. 3, LBMI individuals showed significantly lower levels of G-CSF (GM of 91.1 pg/ml Vs 107.9 pg/ml), GM-CSF (GM of 126.2 pg/ml Vs 174.2 pg/ml), MCP-1 (GM of 119 pg/ml Vs 220.6 pg/ml), MIP-1β (GM of 60.9 pg/ml Vs 86.3 pg/ml), IL-6 (GM of 38.7 pg/ml Vs 72.2 pg/ml), IL-8 (GM of 74.8 pg/ml Vs 205.5 pg/ml) and IL-12p70 (GM of 95.9 pg/ml Vs 107.1 pg/ml) when compared to NBMI individuals. Hence, malnutrition with T2DM is linked to lower circulating levels of pro-inflammatory cytokines.

### Relationship between HbA1c, RBG, pancreatic hormones, adipocytokines, cytokines and BMI

**Fig. 4:**
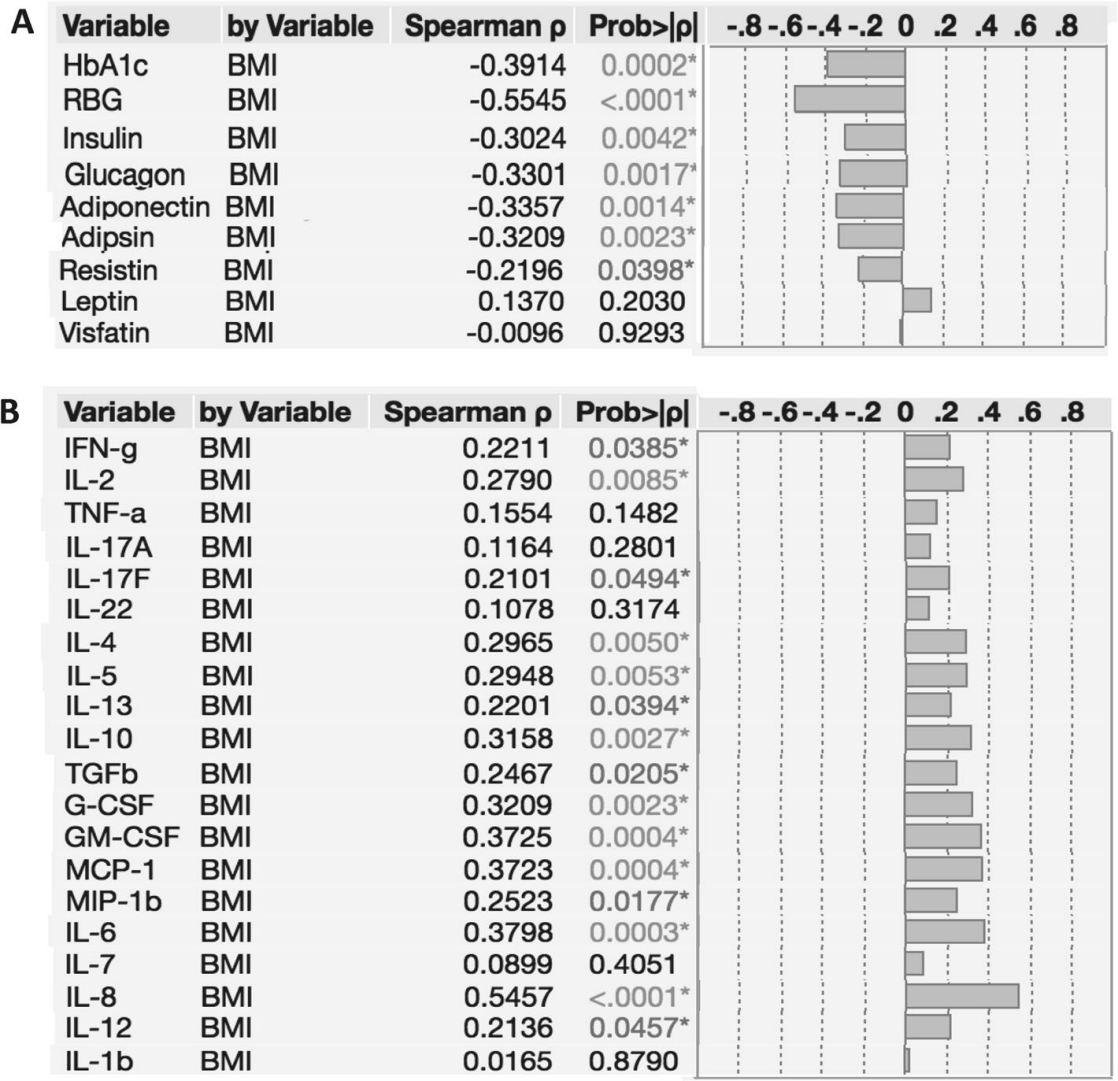
Positive and negative relationship between pancreatic hormones, and cytokine levels and BMI indices in T2DM individuals. The relationship between the (**a**) Plasma levels of glycated haemoglobin (HbA1c), random blood glucose (RBG), insulin and glucagon and BMI indices were examined in all LTBI (*n* = 88) participants. **b** Plasma levels of Type 1 (IFNγ, TNFα and IL-2), Type 17 (IL-17A, IL-17F and IL-22), Type 2 (IL-4, IL-5 and IL-13), regulatory (IL-10 and TGF-β) cytokines and pro-inflammatory (G-CSF, GM-CSF, MCP-1, MIP-1β, IL-6, IL-7, IL-8, IL-12p70 and IL-1β) cytokines and BMI indices were examined in all LTBI (n = 88) participants. The data are represented correlation rank matrices with r values being denoted by horizontal bars. p and *r* values were calculated using the Spearman rank correlation test at 95% confidence intervals using JMP software

To examine the relationship between glycemic parameters, pancreatic hormones, adipocytokines and BMI, we assessed the association of these parameters with BMI in all the individuals in the study. As shown in Fig. 4a, Tables 3 and 4, HbA1c, RBG, insulin and glucagon exhibited a significant negative relationship with BMI, while leptin exhibited a significant positive relationship to BMI. To examine the relationship between the systemic levels of Type 1, Type 17, Type 2, regulatory and other pro-inflammatory cytokines with BMI, we assessed the association of the cytokines with BMI in all the individuals in the study. As shown in Fig. 4b, the systemic levels of IFNγ, IL-2, IL-17F, IL-4, IL-5, IL-13, IL-10, TGF-β, G-CSF, GM-CSF, MCP-1, MIP-1β, IL-6, IL-8 and IL-12p70 each exhibited a significant positive association with BMI. In addition, we have shown the Spearman rank correlation and power analysis to demonstrate the relationship of BMI with biochemical parameters and cytokines and the correlation analysis explicitly shows sufficient correlation between the parameters (Table 3). Power analysis revealed that biochemical parameters have impact on BMI in T2DM individuals (Tables 3 and 4). Thus, biochemical and immunological parameters exhibit a complex relationship to BMI in T2DM individuals.

**Table 3:** Correlation of factors with Body Mass Index (BMI)

	Correlation	***p*** Value
HbA1c	−0.301	0.004
RBG (mg/dl)	−0.587	< 0.001
Insulin (pg/ml)	−0.360	< 0.001
Glucagon (pg/ml)	−0.382	< 0.001
Adiponectin (pg/ml)	−0.355	< 0.001
Adipsin (pg/ml)	−0.331	0.002
Resistin (pg/ml)	−0.178	0.097
Leptin (pg/ml)	0.119	0.269
Visfatin (pg/ml)	−0.018	0.867
IFN-γ (pg/ml)	0.330	0.002
IL-2 (pg/ml)	0.274	0.010
TNF-α (pg/ml)	0.221	0.039
IL-17A (pg/ml)	0.224	0.036
IL-17F (pg/ml)	0.196	0.067
IL-22 (pg/ml)	0.073	0.499
IL-4 (pg/ml)	0.268	0.011
IL-5 (pg/ml)	0.266	0.012
IL-13 (pg/ml)	0.250	0.019
IL-10 (pg/ml)	0.284	0.007
TGF-β (pg/ml)	0.250	0.019
GM-CSF (pg/ml)	0.248	0.020
G-CSF (pg/ml)	0.210	0.049
MCP-1 (pg/ml)	0.342	0.001
MIP-1β (pg/ml)	0.270	0.011
IL-6 (pg/ml)	0.379	< 0.001
IL-7 (pg/ml)	0.206	0.054
IL-8 (pg/ml)	0.444	< 0.001
IL-12 (pg/ml)	0.241	0.024
IL-1β (pg/ml)	0.003	0.975

Spearman rank correlation

**Table 4:** Factors associated with Body Mass Index (BMI)

	Normal BMI	Low BMI	***p***Value
Age in Years	39 (33.5–45)	37 (31–43.5)	0.270
Gender [Male]	22 (50%)	22 (50%)	> 0.950
Albumin (g/dl)	4.3 (3.9–4.5)	3.1 (2.9–3.2)	< 0.001
Urea (mg/dl)	20 (17–22)	22.5 (18.5–26)	0.068
Creatinine (mg/dl)	0.9 (0.7–1)	0.9 (0.8–1)	0.280
ALT (U/L)	20 (17–26)	25 (18–31)	0.204
AST (U/L)	29 (21.5–36)	27 (21.5–42)	> 0.950
Serum triglycerides (mg/dl)	89.5 (75–131)	97.5 (79–154)	0.155
HDL (ml/dl)	35.5 (31–43)	41 (33.5–47)	0.162
LDL (ml/dl)	100 (92–125)	104 (85.5–136)	0.858
RBG (mg/dl)	207 (200–233.5)	271 (224–300)	< 0.001
HbA1c	8.7 (7.5–11.3)	11.7 (9.7–13.3)	< 0.001
Insulin (pg/ml)	52.2 (31.3–90)	82.8 (44.1–187.2)	0.002
Glucagon (pg/ml)	167.9 (150.9–215.8)	250.1 (185–289.7)	< 0.001
Adiponectin (pg/ml)	160,974 (65794–303,991.3)	266,212.7 (96,521.4–451,000.8)	0.023
Adipsin (pg/ml)	17,841.1 (6995.9–45,530.8)	29,648 (12,397.1–71,191.6)	0.021
Resistin (pg/ml)	2437.2 (1539.6–4154)	2828.6 (1988.6–4534.2)	0.374
Leptin (pg/ml)	2003.2 (1605.1–4767.4)	1633.6 (1094.6–2819.1)	0.032
Visfatin (pg/ml)	2978.5 (1660.1–4881.3)	3648.7 (1949.3–4162.3)	0.710
IFN-γ (pg/ml)	372.4 (312.4–410.1)	330.8 (267.2–372.4)	0.004
IL-2 (pg/ml)	173.9 (128.1–219.5)	152.8 (82.4–171.2)	0.010
TNF-α (pg/ml)	318.4 (245–416.2)	265 (211.5–336.5)	0.032
IL-17A (pg/ml)	35.3 (29.2–47.7)	25.5 (18.6–53.8)	0.026
IL-17F (pg/ml)	186 (85.4–238.6)	128.3 (82.9–164.3)	0.041
IL-22 (pg/ml)	97.8 (62–129)	70.1 (37.5–125.2)	0.243
IL-4 (pg/ml)	49 (31.5–128.8)	33.5 (31.1–35.5)	0.006
IL-5 (pg/ml)	120.6 (114.3–126.9)	96.9 (71.1–124.3)	0.014
IL-13 (pg/ml)	78.2 (43.4–98)	33.3 (22.4–81.9)	0.008
IL-10 (pg/ml)	111.4 (102.1–119.2)	96 (90–111.4)	< 0.001
TGF-β (pg/ml)	78.2 (43.4–98)	33.3 (22.4–81.9)	0.008
GM-CSF (pg/ml)	206.9 (154.4–252.8)	151.3 (74.5–220.1)	0.015
G-CSF (pg/ml)	111.4 (99.9–127.5)	95.4 (73–109.1)	0.001
MCP-1 (pg/ml)	234.8 (175.7–327.7)	165.4 (72.1–247.3)	< 0.001
MIP-1β (pg/ml)	102 (38.9–208.3)	65.3 (49.5–79.5)	0.047
IL-6 (pg/ml)	78.3 (49.2–121.1)	38.1 (26.6–48.8)	< 0.001
IL-7 (pg/ml)	58.2 (49.4–98.2)	64.5 (32.4–85.5)	0.341
IL-8 (pg/ml)	284.6 (145.6–322.3)	79.1 (42.6–134.6)	< 0.001
IL-12 (pg/ml)	98.2 (90.1–118.2)	90.1 (82.2–104.9)	0.026
IL-1β (pg/ml)	66.8 (50–125.3)	69.4 (54.4–74.7)	0.455

Mann Whitney U test was used at 5% level of significance

### Principle component analysis reveals trends in pancreatic hormones, adipocytokines, cytokines and BMI

**Fig. 5:**
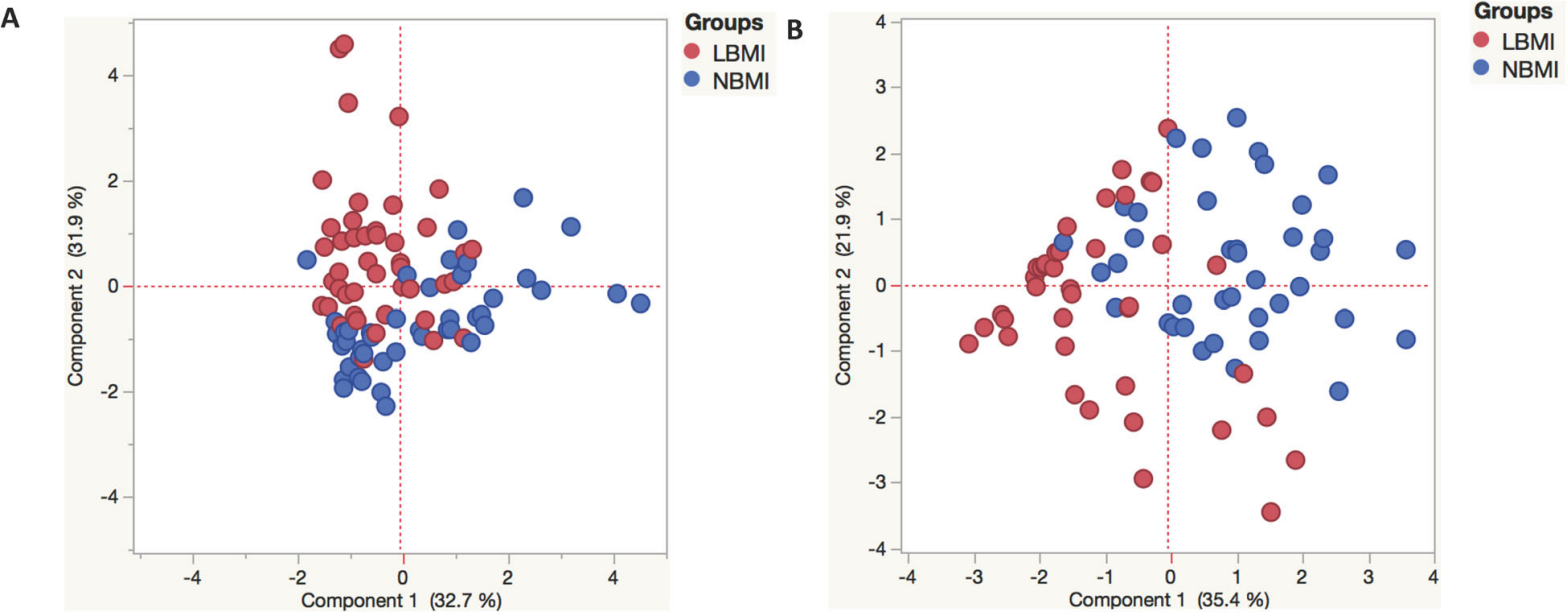
Principle component Analysis (PCA) depicting circulating levels of pancreatic hormones and cytokines in LBMI and NBMI individuals. Principal component analysis (PCA) was done to exhibit the dissemination of data from the mixture of two groups LBMI (blue circles) and NBMI (red circles). The PCA indicates the two principal components of variation. **a** PCA analysis was done with pancreatic hormone and adipocytokine between LBMI and NBMI participants with T2DM. **b** PCA analysis of cytokines between LBMI and NBMI participants

PCA was performed to envision variation between the groups created on the complete data set. To envision the clustering pattern of pancreatic hormones, adipocytokines and cytokines between LBMI (red circle) and NBMI (blue circle) individuals, we plotted PCA with pancreatic hormones (insulin and glucagon) and adipocytokines (adiponectin, adipsin, resistin, leptin, visfatin). In addition, the levels of Type 1 cytokines: IFNγ, TNFα, IL-2, Type 17 cytokines: IL-17A, IL-17F, IL-22, Type 2 cytokines: IL-4, IL-5, IL-13, regulatory cytokines: IL-10, TGF-β and pro-inflammatory cytokines: G-CSF, GM-CSF, MCP-1, MIP-1β, IL-6, IL-7, IL-8, IL-12 and IL-1β) were plotted between the 2 groups. The factors were reduced by excluding the factors with the commonalities as low as 0.3 and then the revised PCA was presented in the article with the following factors, PCA-1 (adiponectin, adipsin, Glucagon and insulin) and PCA-2 (Glucagon, insulin and HbA1c). The above factors were used and the plot of first two components was presented with its variability. As illustrated in Fig. 5a, PCA analysis showed that pancreatic hormone and adipocytokine clusters varied between LBMI and NBMI individuals with T2DM. The score plot of the first two components revealed 32.7 and 31.9% of overall variance, respectively. As illustrated in Fig. 5b, PCA analysis of cytokines showed two different clusters between LBMI and NBMI individuals. The factors were reduced by excluding the factors with the commonalities as low as 0.3 and then the revised PCA was presented using following factors, PCA-1 (TGF-β, GM-CSF, IL-13, IL-2, IL-17F, IL-4 and IL-8) and PCA-2 (GM-CSF, IL-2, IL-17F and IL-8). The above factors were used and the plot of first two components was presented with its variability. The score plot of the first two components revealed 35.4 and 21.9% of overall variance, respectively. The findings of PCA results were similar with multivariate and univariate analysis. Thus, PCA analysis divulge the overall influence of pancreatic hormones, adipocytokines and cytokine of T2DM individuals on BMI.

### Multivariate regression and power analysis of T2DM and BMI

In this study, multivariate regression analysis was performed to determine the impact of confounding variables on the numerous analytes determined in T2DM individuals. As presented in Table 5, the levels of biochemical parameters such as AST, Serum triglycerides, HDL, LDL, RBG, HbA1c, diabetic parameters such as insulin, glucagon, adiponectin, adipsin, resistin, leptin and visfatin; cytokines like IFNγ, TNFα, IL-2, IL-17A, IL-17F, IL-22, IL-4, IL-5, IL-13, IL-10, TGF-β and pro-inflammatory cytokines: G-CSF, GM-CSF, MCP-1, MIP-1β, IL-6, IL-7, IL-8, IL-12p70 and IL-1β and were all significantly influenced by BMI even after adjusting for the effect of the age and gender. Hence, our data corroborate that LBMI has an impact upon numerous relevant crucial parameters in T2DM individuals, including blood glucose levels, and the levels of the adipocytokines and the more conventional cytokines.

**Table 5:** Factors associated with Lower Body Mass Index (BMI)

	OR (95% CI)	***p*** Value	aOR (95% CI)	***p*** Value
Age in Years	0.97 (0.93–1.02)	0.231		
Gender [Male]	1.00 (0.43–2.31)	> 0.950		
Urea (mg/dl)	1.98 (0.74–5.30)	0.176	1.83 (0.67–5.00)	0.236
Creatinine (mg/dl)	3.42 (0.57–20.58)	0.18	3.21 (0.51–20.07)	0.213
ALT (U/L)	1.49 (0.74–2.99)	0.262	1.45 (0.71–2.95)	0.311
AST (U/L)	1.14 (0.64–2.03)	0.651	1.12 (0.62–2.01)	0.711
Serum triglycerides (mg/dl)	1.43 (0.80–2.56)	0.227	1.63 (0.87–3.06)	0.125
HDL (ml/dl)	1.70 (0.56–5.15)	0.348	1.78 (0.58–5.50)	0.316
LDL (ml/dl)	1.12 (0.43–2.92)	0.816	1.13 (0.43–2.97)	0.81
RBG (mg/dl)	92.36 (9.17–929.76)	< 0.001	91.26 (9.03–922.12)	< 0.001
HbA1c	14.40 (3.43–60.53)	< 0.001	15.66 (3.61–67.88)	< 0.001
Insulin (pg/ml)	1.94 (1.27–2.94)	0.002	1.93 (1.25–2.97)	0.003
Glucagon (pg/ml)	5.33 (2.00–14.21)	< 0.001	5.17 (1.92–13.89)	0.001
Adiponectin (pg/ml)	1.44 (1.04–1.98)	0.026	1.44 (1.04–1.99)	0.027
Adipsin (pg/ml)	1.44 (1.10–1.90)	0.009	1.52 (1.14–2.05)	0.005
Resistin (pg/ml)	1.28 (0.78–2.10)	0.332	1.33 (0.80–2.21)	0.272
Leptin (pg/ml)	0.60 (0.38–0.96)	0.035	0.59 (0.37–0.95)	0.031
Visfatin (pg/ml)	1.05 (0.69–1.60)	0.821	1.01 (0.66–1.55)	> 0.950
IFN-γ (pg/ml)	0.21 (0.06–0.68)	0.009	0.19 (0.06–0.64)	0.007
IL-2 (pg/ml)	0.37 (0.18–0.77)	0.008	0.35 (0.16–0.75)	0.007
TNF-α (pg/ml)	0.31 (0.12–0.79)	0.015	0.32 (0.13–0.84)	0.02
IL-17A (pg/ml)	0.55 (0.31–0.97)	0.04	0.57 (0.32–1.02)	0.057
IL-17F (pg/ml)	0.59 (0.33–1.06)	0.078	0.58 (0.32–1.05)	0.073
IL-22 (pg/ml)	0.71 (0.43–1.17)	0.177	0.64 (0.38–1.08)	0.095
IL-4 (pg/ml)	0.18 (0.07–0.46)	< 0.001	0.18 (0.07–0.46)	< 0.001
IL-5 (pg/ml)	0.16 (0.05–0.52)	0.003	0.17 (0.05–0.56)	0.004
IL-13 (pg/ml)	0.52 (0.32–0.82)	0.005	0.48 (0.29–0.78)	0.003
IL-10 (pg/ml)	0.06 (0.01–0.46)	0.006	0.05 (0.01–0.39)	0.004
TGF-β (pg/ml)	0.52 (0.32–0.82)	0.005	0.48 (0.29–0.78)	0.003
GM-CSF (pg/ml)	0.55 (0.33–0.92)	0.022	0.55 (0.33–0.93)	0.024
G-CSF (pg/ml)	0.19 (0.06–0.62)	0.006	0.19 (0.06–0.67)	0.009
MCP-1 (pg/ml)	0.41 (0.24–0.71)	0.001	0.41 (0.23–0.71)	0.002
MIP-1β (pg/ml)	0.64 (0.42–0.97)	0.034	0.65 (0.43–0.99)	0.045
IL-6 (pg/ml)	0.25 (0.14–0.48)	< 0.001	0.24 (0.13–0.46)	< 0.001
IL-7 (pg/ml)	0.60 (0.34–1.07)	0.085	0.57 (0.31–1.06)	0.076
IL-8 (pg/ml)	0.29 (0.18–0.49)	< 0.001	0.28 (0.17–0.48)	< 0.001
IL-12 (pg/ml)	0.36 (0.11–1.13)	0.081	0.39 (0.12–1.27)	0.118
IL-1β (pg/ml)	0.51 (0.26–0.99)	0.048	0.50 (0.25–0.99)	0.048

*OR* Univariate Odds Ratio; *aOR* Multivariate Odds Ratio adjusted for Age and Gender

## Discussion

Epidemiological studies have shown that while there is a relationship between increased BMI and T2DM risk in all race/ethnic populations, individuals of Asian descent tend to develop T2DM at a much lower BMI than other race/ethnic groups [22–24]. Indeed, several recent studies have described a high prevalence of T2DM in normal weight Asian individuals [25–27]. Understanding the biochemical and immunological features of T2DM in non-obese individuals would provide insights into the connection between insulin resistance and Type 2 DM risk and reveal novel aspects of disease pathophysiology. However, very few studies have been conducted in this subset of individuals. Our study is one of the first to explore the underlying biochemical and immunological alterations associated with this low BMI and T2DM interaction.

We first explored the influence of low BMI on parameters associated with glycemic control in T2DM individuals. Our data clearly reveal impact of undernutrition on glycemic indices and pancreatic hormones. Thus, LBMI is associated with higher levels of HbA1c, RBG, insulin and glucagon. Our data highlight the fact that glycemic control is poorer in LBMI than NBMI individuals, indicating a detrimental effect of LBMI on metabolic disease. Previous studies from China and Japan suggested that LBMI in T2DM might be typically associated with low insulin levels [19, 28]. In contrast, our data suggest that the LBMI in the South Indian population is actually associated with higher levels of insulin and glucagon. Since, both insulin and glucagon are associated with a catabolic metabolic state, this suggests that higher levels of these pancreatic hormones could be potential drivers of loss of weight and lower plasma cytokines in T2DM individuals. In addition, our data also imply that despite higher insulin levels, hyperglycemia is greater in LBMI individuals with T2DM suggesting a phenotype of insulin resistance in these individuals. An imbalance between pro- and anti-inflammatory adipokines could also contribute to the development of insulin resistance [29]. Adipose tissue from lean individuals predominantly secretes anti-inflammatory adipokines such as adiponectin and adipsin. In contrast, adipose tissue of obese individuals predominantly secretes pro-inflammatory adipokines including leptin, resistin and visfatin [30]. In agreement with this, our data also reveal higher levels of adiponectin and adipsin and lower levels of leptin in LBMI individuals with T2DM. Thus, low BMI is associated with modulation of the levels of adipokines.

Malnutrition is associated with modulation of immune responses, including those associated with innate and adaptive responses [15]. Undernutrition is typically associated with deficiencies in the production of pro-inflammatory cytokines and upregulation of regulatory cytokines [12]. The imbalance among the cytokine milieu in LBMI individuals could due to lack of energy and building blocks to synthesize the proteins needed or it could be due to reduce in thymus size and reduced Delayed Type Hyper Sensitivity (DTHR) [16]. In our study, both Type 1(IFNγ, TNFα and IL-2) and Type 17 (IL-17A and IL-17F) cytokines were significantly lower in LBMI individuals than in NBMI individuals with T2DM. Our data also reveal significantly lower circulating levels of pro-inflammatory cytokines – G-CSF, GM-CSF, MCP-1, MIP-1β, IL-6, IL-8 and IL-12p70 – in LBMI individuals with T2DM. Thus, our data suggest that a mechanistic underpinning of the undernutrition – diabetes interface, involving the modulation of Type 1, Type 17 and other pro-inflammatory cytokine responses, that has the propensity for altering insulin sensitivity and promoting an impaired ability to respond to pathogenic insults. Typically, undernutrition is associated with an upregulation of Type 2 and regulatory cytokines [12, 16]. However, our data in LBMI with T2DM individuals suggests that even the production of Type 2 and regulatory cytokines at homeostasis is altered by the metabolic interaction between BMI and DM. Since Type 2 and regulatory cytokines are associated with improved insulin sensitivity, our data provide an additional mechanism by which LBMI influences T2DM, i.e. by inducing alterations in the cytokine milieu of Type 2 and regulatory cytokines. Lower production of both pro- and anti-inflammatory cytokines has potentially troubling connotations, including increased susceptibility to infection and inability to resolve inflammatory events. Thus, low BMI and T2DM could potentially create a synergistic milieu leading to deleterious consequences for host immune responses.

Finally, our data on the association between different parameters and BMI reveals certain interesting features. Firstly, glycemic parameters and pancreatic hormones exhibit a negative correlation with BMI and so do adipokines, with the exception of leptin. Secondly, almost all the immune parameters (cytokines) examined exhibit a positive correlation with BMI suggesting that lower BMI is directly associated with lower plasma cytokine levels. Hence, nutritional replenishment of low BMI individuals would be a simple but important method to restore normal immune responses in these individuals irrespective of the presence of T2DM. Whether the presence of lower levels of pro-inflammatory cytokines and adipokines actually translates to a dampened inflammatory milieu in T2DM remains to be determined. Similarly, the relationship between higher insulin and glucagon levels and the impaired plasma cytokine levels also needs to be examined. Thirdly, our data on haematological parameters in LBMI or NBMI with T2DM suggests that alterations in the cytokine milieu is not due to perturbations in innate or adaptive cell numbers or frequencies since complete blood and differential counts are not significantly different between the two groups.

Our study has several limitations. We could not correct for all possible confounding variables as we did not measure fat mass, mid upper arm circumference, waist circumference etc. Moreover, being a cross-sectional study, we could not draw any inferences on cause and effect. We have limited the measurement to only the circulating levels of adipokines and cytokines and not the cellular levels. Also, we have not measured the immunological markers for Type1 diabetes with low sample size. However, our study demonstrates that metabolic dysfunction due to disparity in the expression levels of pro- and anti-inflammatory adipocytokines is associated with the severity of T2DM. Although, adipocytokines may function as regulators of body homeostasis, our data implies that these parameters are altered by under nutrition in people living with T2DM. This could suggest that nutritional impacts exerted by LBMI could shape clinical outcomes of T2DM. Thus, additional elucidation of the functions and mechanisms of adipocytokines and pro and anti-inflammatory cytokines will lead to an improved understanding of the pathogenesis of metabolic disorders associated with nutrition.

## Conclusion

Our study thus provides important insights into the immunological interaction between undernutrition and diabetes mellitus. Our data suggests that undernutrition has the potential to make diabetes worse, promote insulin resistance, alter the balance of adipocytokines and dampen host protective cytokine responses. Our study also reveals the necessity to explore the immunological profiles of low BMI in other metabolic disorders. Unravelling a common immunological or biochemical pathway by which low BMI impacts metabolic disease in general would be beneficial in coming up with host – directed strategies to combat this harmful interaction. Finally, longitudinal studies with nutritional replenishment is necessary to determine the direct impact of proper nutrition in restoring immunological and biochemical balance in diabetes mellitus.

## Supplementary Information

**Additional file 1:** **Figure S1.** Flowchart for the study participant recruitment. A flowchart illustrating participant recruitment to the study.

**Additional file 2:** **Table S1.**

## Data Availability

All data generated or analysed during this study are included in this published article [and its supplementary information files]. “The datasets used and/or analysed during the current study are available from the corresponding author on reasonable request.”

## Abbreviations

BMI: Body Mass Index
LBMI: Low Body Mass Index
NBMI: Normal Body Mass Index
HbA1c: Glycated haemoglobin
RBG: Random Blood Glucose
