# Supplementary figures and images for: Impact of malnutrition on systemic immune and metabolic profiles in type 2 diabetes
Source: BMC Endocr Disord. 2020 Nov 12;20:168. doi: 10.1186/s12902-020-00649-7 (PMC7659078; doi:10.1186/s12902-020-00649-7)

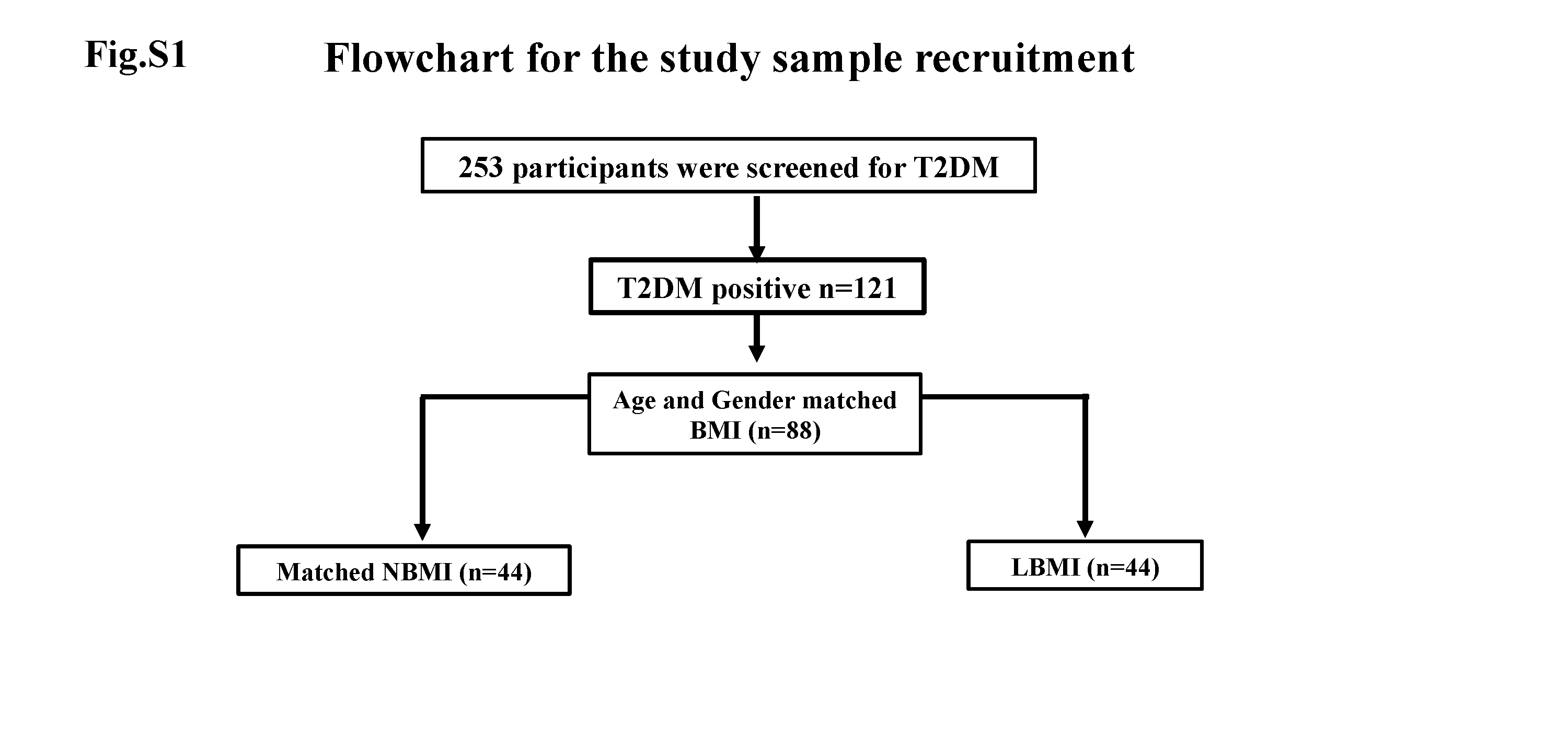

Supplement: Supplementary file 1 — Additional file 1: Figure S1. Flowchart for the study participant recruitment. A flowchart illustrating participant recruitment to the study. [file 12902_2020_649_MOESM1_ESM.tiff]
