# Supplementary material for: Impact of malnutrition on systemic immune and metabolic profiles in type 2 diabetes
Source: BMC Endocr Disord. 2020 Nov 12;20:168. doi: 10.1186/s12902-020-00649-7 (PMC7659078; doi:10.1186/s12902-020-00649-7)
Supplement: Supplementary file 2 — Additional file 2: Table S1. [file 12902_2020_649_MOESM2_ESM.docx]

| **Table S1** |  |  | **LBMI** | | | **NBMI** | | |
| --- | --- | --- | --- | --- | --- | --- | --- | --- |
| **Parameters and Detection limit** | **inter-assay coefficient of variation (CV%)** | **intra-assay coefficient of variation (CV%)** | **Mean** | **SD** | **%CV** | **Mean** | **SD** | **%CV** |
| **HbA1c** |  |  | 11.46 | 2.02 | 0.18 | 9.55 | 2.78 | 0.29 |
| **RBG** |  |  | 290.00 | 86.60 | 0.30 | 223.90 | 50.40 | 0.23 |
| **BMI** |  |  | 14.86 | 1.94 | 13.02% | 22.10 | 1.35 | 22.51% |
| **Insulin**  **(1.7- 3,541 pg/ml)** |  |  | 140.20 | 134.50 | 95.92% | 68.77 | 52.39 | 76.18% |
| **Glucagon**  **15.7 - 3,500 pg/ml** |  |  | 247.60 | 88.35 | 35.69% | 185.80 | 59.01 | 31.75% |
| **Adiponectin**  **160 - 218,485 pg/ml** | 2 |  | 308105.00 | 241068.00 | 78.24% | 196930.00 | 156337.00 | 79.39% |
| **Adipsin**  **43 - 14,513 pg/ml** | 4 |  | 47715.00 | 46688.00 | 97.85% | 27168.00 | 25481.00 | 93.79% |
| **Resistin**  **2.3 - 4,739 pg/ml** | 4 |  | 3162.00 | 1531.00 | 48.41% | 2959.00 | 1765.00 | 59.65% |
| **Leptin**  **11.5 - 129,107 pg/ml** | 4 |  | 2172.00 | 1418.00 | 65.30% | 3091.00 | 1956.00 | 63.30% |
| **Visfatin**  **51.3 - 280,266 pg/ml** | 3 |  | 3240.00 | 1479.00 | 45.64% | 3461.00 | 2319.00 | 67.00% |
| **IFN-γ**  **92.6−52,719 pg/ml** | 9 | 15 | 314.10 | 83.98 | 26.74% | 368.20 | 86.43 | 23.47% |
| **TNF-α**  **5.8− 95,484 pg/ml** | 6 | 8 | 274.00 | 88.73 | 32.39% | 323.70 | 92.92 | 28.70% |
| **IL-2**  **2.2- 17,772 pg/ml** | 9 | 7 | 140.90 | 64.38 | 45.69% | 172.10 | 53.41 | 31.03% |
| **IL-17A**  **4.7-12,235 pg/ml** | 6 | 8 | 36.67 | 23.70 | 64.63% | 41.67 | 18.79 | 45.09% |
| **IL-17F**  **12.5 - 800 pg/mL** | 7 | 4.1 | 132.20 | 54.31 | 41.09% | 169.30 | 82.88 | 48.95% |
| **IL-22**  **2.5-41,572 pg/ml** | 7 | 8 | 80.40 | 43.85 | 54.54% | 91.59 | 40.75 | 44.49% |
| **IL-4**  **2.2- 3,467 pg/ml** | 8 | 8 | 33.20 | 5.27 | 15.87% | 82.63 | 62.15 | 75.21% |
| **IL-5**  **3.1- 7,380 pg/ml** | 10 | 8 | 96.55 | 32.81 | 33.98% | 120.80 | 25.88 | 21.42% |
| **IL-13**  **3.7- 3,137 pg/ml** | 7 | 8 | 57.65 | 45.45 | 78.84% | 75.21 | 35.17 | 46.76% |
| **IL-10**  **2.2- 8,840 pg/ml** | 6 | 5 | 97.66 | 16.11 | 16.50% | 109.90 | 18.44 | 16.78% |
| **TGF-β**  **31.2-2,000 pg/ml** | 6.4 | 2.5 | 177.50 | 90.57 | 51.03% | 241.30 | 113.90 | 47.22% |
| **G-CSF**  **2.4- 11,565 pg/ml** | 5 | 10 | 94.50 | 26.12 | 27.65% | 111.20 | 24.90 | 22.40% |
| **GM-CSF**  **63.3- 6,039 pg/ml** | 6 | 12 | 152.80 | 80.89 | 52.92% | 194.50 | 73.02 | 37.53% |
| **MCP-1**  **2.1- 1,820 pg/ml** | 7 | 9 | 164.10 | 106.90 | 65.15% | 241.10 | 86.23 | 35.77% |
| **MIP-1β**  **2.0- 1,726 pg/ml** | 8 | 7 | 65.31 | 22.51 | 34.46% | 126.90 | 97.78 | 77.05% |
| **IL-6**  **2.3- 18,880 pg/ml** | 11 | 7 | 43.29 | 24.34 | 56.23% | 84.95 | 44.61 | 52.52% |
| **IL-7**  **3.1- 6,001 pg/ml** | 8 | 6 | 64.13 | 32.18 | 55.68 | 76.36 | 40.96 | 53.64% |
| **IL-8**  **1.9- 26,403 pg/ml** | 4 | 9 | 99.33 | 77.28 | 77.81% | 244.50 | 121.50 | 49.71% |
| **IL-12P70**  **3.3-13,099 pg/ml** | 6 | 6 | 99.17 | 28.21 | 28.45% | 113.50 | 49.02 | 43.20% |
| **IL-1β**  **3.2- 3,261 pg/ml** | 8 | 6 | 64.66 | 17.98 | 27.80% | 90.68 | 59.12 | 65.20% |

**SD -Standard Deviation**

**CV -Coefficient of variation**
